# Effects of Varying Light Durations on Sperm Quality in Rams

**DOI:** 10.3390/ani14243592

**Published:** 2024-12-12

**Authors:** Zhendong Zhu, Wenjia Li, Haolong Zhao, Adedeji Olufemi Adetunji, Ahmed Mohamed Kamel, Lingjiang Min

**Affiliations:** 1 College of Animal Science and Technology, Qingdao Agricultural University, Qingdao 266109, China; zzd2020@qau.edu.cn (Z.Z.); 20222203015@stu.qau.edu.cn (W.L.); zhaohaolong@stu.qau.edu.cn (H.Z.); 2 Department of Agriculture, University of Arkansas at Pine Bluff, Pine Bluff, AR 71601, USA; adetunjiadedeji.aa@gmail.com; 3 Animal and Poultry Production Division, Desert Research Center, Mataria, Cairo 11753, Egypt; ahmed_kamel_drc@yahoo.com

**Keywords:** light, sperm, metabolome, ram, seminal plasma

## Abstract

This investigation aimed to study the effects of varying light exposure durations on ram sperm. A total of 25 rams were randomly divided into five groups. The control group was exposed to light durations of 12 h, while the experimental groups were exposed to light durations of 14, 16, 18, and 20 h. After three months of rearing, semen was collected from each ram four times using the artificial vagina method. The sperm motility parameters, sperm abnormality, sperm concentration, acrosome integrity, membrane integrity, semen volume, and total sperm number were measured. Thereafter, the metabolome, amino acid level, testosterone content, plasma follicle-stimulating hormone (FSH) and luteinizing hormone (LH) levels, and sperm antioxidant capacity were measured. The results showed that the sperm motility, sperm concentration, ejaculation volume, total sperm number, acrosome integrity, and membrane integrity in the 16 h light group were significantly improved compared to the control (*p* < 0.05), meanwhile the sperm abnormality was decreased. Moreover, we found 345 different metabolites between the control and 16 h light group. Among these, 273 were upregulated and 72 were downregulated. Furthermore, the amino acid content of the seminal plasma in the 16 h light group was significantly increased (*p* < 0.05) compared to the control. Interestingly, the seminal plasma testosterone content and the levels of FSH and LH in the serum in the 16 h light group were significantly increased (*p* < 0.05) compared to the control. In terms of the sperm antioxidant capacity, it was observed that the CAT activity was the highest in the group exposed to 16 h of light and decreased at 18 h of light exposure when compared to the control group; however, the CAT activity at 20 h was not different from the control. Additionally, within the 14 to 18 h light exposure range, prolonged light exposure increased the GSH content (*p* < 0.05), whereas 20 h of light exposure reduced the GSH content. The MDA levels decreased with prolonged light exposure, reaching the lowest point at 16 h (*p* < 0.05), but increased again at 20 h of light exposure. KEGG analysis indicated that the differential metabolites were mainly involved in metabolic and synthetic activities. Based on the results of this study, we can conclude that the artificial extension of the light duration for 16 h has a positive effect on ram sperm quality.

## Simple Summary

In this study, 25 rams were randomly divided into five groups to investigate the effects of different photoperiods on the ram sperm. The control group was exposed to 12 h of light, while the experimental groups were exposed to 14, 16, 18, and 20 h of light. The results showed that compared to the control group, the group exposed to 16 h of light had significantly improved sperm motilities, sperm concentrations, ejaculate volumes, total sperm counts, sperm abnormalities, acrosome integrities, and membrane integrities (*p* < 0.05). Additionally, we identified 345 different metabolites between the control and 16 h light group, with 273 upregulated and 72 downregulated. The amino acid content of the seminal plasma was significantly higher in the 16 h light group compared to the control (*p* < 0.05). Furthermore, compared to the control group, the 16 h light group exhibited significantly higher levels of seminal plasma testosterone, serum follicle-stimulating hormone (FSH), and luteinizing hormone (LH) (*p* < 0.05). In terms of the sperm antioxidant capacity, the catalase (CAT) activity was the highest in the 16 h light group. Additionally, prolonged light exposure between 14 and 18 h increased the glutathione (GSH) levels (*p* < 0.05), and the malondialdehyde (MDA) levels reached their lowest point at 16 h of light exposure (*p* < 0.05) but increased again with 20 h of light. Overall, the artificial extension of the photoperiod to 16 h had a positive effect on the ram sperm quality.

## 1. Introduction

Artificial insemination (AI) not only facilitates genetic enhancement and reproductive management but also preserves invaluable genetic data [1]. This underscores the undeniable significance of AI in the small ruminant industry [2]. However, the morphology of the sheep’s cervix poses limitations on AI in sheep [3]. Therefore, ensuring the colonization and migration of semen through the cervix is contingent upon high-quality semen used in sheep AI. In addition, many reports have confirmed that the vitality and quality of the semen are closely related to the fertility of the sheep [4,5,6].

The mechanism influencing the semen quality in small ruminants, such as sheep, is intricate. For instance, the small ruminant reproductive system is influenced by environmental factors and nutritional signals [7]. Notably, Guan et al. [8] reported that malnutrition-induced testicular degeneration and sperm DNA damage in sexually mature male sheep resulted in decreased spermatogenesis efficiency and impaired sperm motility [8]. Furthermore, improved spermatogenic genes (*Sycp9*, *TDRD9*, *BRDT*, *CEP120*, *BRCA*) enhanced the protection of normal sperm production in heat-stressed sheep [9]. Additionally, exposure to heavy metals from soil near thermal power plants significantly compromised the endocrine function and reproductive potential of sheep born and raised in these areas specifically within their testes [10]. Moreover, the photoperiod impacts the quality of sheep sperm significantly, since sheep are a seasonal breed.

Recently, there has been significant interest in understanding the light influence on semen quality. Yeste et al. [11] demonstrated that light irradiation can enhance sperm motility and prolong sperm survival without causing damage to the sperm DNA integrity [11]. Notably, the duration of light exposure plays a crucial role in sheep breeding outcomes [12]. Hurley et al. reported that artificial photoperiods improved guinea pig sperm characteristics and facilitated precocious reproduction, while natural solar cycles resulted in reduced sperm quality and impaired fertility due to heat stress-induced infertility [13]. Moreover, prolonged exposure to light increased the testicular and seminal vesicle size in voles [14]. Surprisingly, the influence of seasonal variations on the semen quality in Mexico was effectively mitigated by precisely controlling for both long and short days, while ensuring no alteration in the sperm fertility [15]. In addition, it has been documented that these alterations in photoperiods exert an influence on the characteristics of semen and seminal plasma [16]. Therefore, it is necessary to consider the mechanism of the photoperiodic changes affecting the production and quality of sheep sperm.

Seminal plasma, a complex, multiphase fluid primarily secreted by the accessory gonads, constitutes an integral component of semen [17]. Compared to sperm, the seminal plasma exhibits significantly elevated quantities and levels of metabolites. Seminal plasma comprises lipids, amino acids, steroid hormones, and other metabolites [18]. Given that metabolites serve as the ultimate products of metabolic pathways, they exert a pivotal role in sperm physiology by governing energy metabolism, motility, and overall metabolic activity [19]. Research on the effects of the photoperiod on seminal plasma metabolites highlights their significant role in modulating the sperm function and overall reproductive performance in male ruminants [20]. Changes in the photoperiod, which influence reproductive hormones like melatonin, testosterone, and gonadotropins, have been linked to seasonal variations in the seminal plasma composition and sperm quality [21]. A previous study showed that the photoperiod affected the seminal plasma’s protein composition and antioxidant capacity [16]. For example, an increased photoperiod has been associated with higher melatonin levels in seminal plasma, correlating with enhanced antioxidant activity and sperm protection in rams [21,22].

Numerous metabolites present in seminal plasma have demonstrated their potential as fertility markers for predicting reproductive efficiency [20]. The testosterone and estradiol levels in seminal plasma can serve as prognostic indicators for normal spermatogenesis [23]. Relevant research has consistently demonstrated the significant role of trace elements inherent in seminal plasma on the quality of sperm [24,25,26]. Elevated copper levels in sperm exert a pronounced detrimental impact on sperm motility [26]. A recent study demonstrated that disproportionate lead levels in seminal plasma influence the semen quality [27]. Furthermore, the incorporation of lipid constituents such as cholesterol and triglycerides within seminal plasma holds promise for enhancing the assessment of male semen quality [28]. The free amino acids present in seminal plasma serve a multitude of physiological roles, encompassing the mitigation of oxidative stress, the preservation of cellular integrity, and the provision of oxidizable substrates for spermatozoa [29]. The amino acids alanine, glycine, glutamine, histidine, and proline have demonstrated protective effects on spermatozoa by inhibiting lipid peroxidation or regulating osmotic mechanisms [30,31,32]. For instance, proline enhances motility and safeguards sperm cells against free radical-induced damage by stabilizing the structure and function of membranes [33]. Furthermore, alanine enhanced the sperm motility by augmenting the cryoprotective efficacy of glycerol to a certain extent [34].

Artificial insemination technology is becoming more prevalent in the pig, cattle, sheep, and other breed industries. In addition, the mechanism of the photoperiod effect on semen quality has not been fully clarified. In this study, we sought to use high-throughput techniques to examine whether the photoperiod affects the semen quality by altering relevant metabolites in the seminal plasma. This study provides some references for the mechanism of the photoperiod affecting the semen quality. In addition, some specific metabolites obtained can be used as biomarkers to evaluate the semen quality of sheep and predict male fertility.

## 2. Materials and Methods

### 2.1. Animal Care

All animals and experimental procedures were approved by the Qingdao Agriculture University Institutional Animal Care and Use Committee (QAU1121010).

### 2.2. Animal Semen Collection

This study used 25 healthy-fertility adult rams (“HD line sheep” refers to HongDe-line sheep, a highly crossbred strain of the Small-tailed Han sheep, commonly known as the “HongDe line” by locals) aged about 2 years, with an average weight of 80 ± 3.20 kg. The rams with strong sexual desire and good sperm motility (more than 80%) were preselected for the experiment. The rams were randomly divided into five groups. Usually, 12 h of light is considered the threshold for the transition from the short-day to long-day photoperiodism in animals. In this study, we aimed to investigate whether and how the long photoperiod affected the ram sperm quality. Thus, the rams were divided into five groups and were fed under different light durations (12 h, 14 h, 16 h, 18 h, 20 h) with a light intensity of 100 watts (warm white light; wavelength: 2800–3200 nm; brightness: 400–600 mcd). The rams were housed in a dark room with lights turned on at 7:00 AM. For the control group (12 h), lights were turned off at 7:00 PM, while for the treatment groups (14 h, 16 h, 18 h, 20 h), lights were turned off at 9:00 PM, 11:00 PM, 1:00 AM, and 3:00 AM, respectively. Moreover, the barn was equipped with a ventilation system, maintaining a stable temperature of 25 °C and a humidity level of 55% across all groups during the experiment period. Before the experiment, the rams were pretreated for a week according to the experiment conditions. After three months of feeding, 100 ejaculations were obtained using an artificial vagina to measure the sperm motility, concentration, and other parameters. During the ram ejaculation collection, the ram semen collection equipment was strictly disinfected before use. The inner tube of the artificial vagina was disinfected with 75% alcohol and then coated with vaseline. When the ram mounted the ewe and extended the penis, the ejaculation was collected, and then it was immediately taken off the artificial vagina and erected to make the semen flow into the sperm collection cup. The semen was used for the following experimental analysis. The frequency of the semen collection was twice a week. The semen was collected and evaluated by two professional technicians to minimize the intra- and inter-technician variability in this study.

### 2.3. Assessment of Sperm Motility, Semen Volume, Sperm Concentration, and Total Number of Sperm

Sperm motility was analyzed with a computer-assisted sperm analysis (CASA) (SCA 20-06-01; Goldcyto, Barcelona, Spain). A digital camera (acA780-75gc, Basler, Germany, Berlin, 10115-14199) connected to a negative-phase contrast microscope (100× magnification) set to a standard parameter of 25 frames/s was used to acquire images. Prior to detection, 5 µL semen sample aliquots were added to the Maker chamber after the preheating process of the analyzer was completed [35]. Assessment of the sperm motility was conducted in three randomly selected areas. In total, over 500 sperm were evaluated. Total sperm motility denotes the percentage of sperm moving at a path speed of 12 µm/s. Moreover, sperm moving at a path velocity of 45 µm/s for over 80% of the period in a straight line was defined as forward movement. A hemocytometer and a graduated collection tube was used to measure the sperm concentration and semen volume, respectively. To derive the total number of spermatozoa or ejaculates, the volume of the ejaculate was multiplied by the concentration of sperm/mL. Analyses were carried out in triplicate.

### 2.4. Evaluation of Sperm Abnormalities

In brief, to evaluate the sperms’ abnormalities, 10 μL of semen was immobilized with 0.5 μL (4%) of paraformaldehyde and stained with an eosin staining solution [36]. Moreover, stained sperm were monitored under a 200× magnification microscope (ZEISS DM200LED, Oberkochen, Germany) [37]. Abnormalities in the sperm, including abnormalities in the head, middle, and tail of the sperm, were recorded. Analyses were carried out in triplicate.

### 2.5. Evaluation of Sperm Acrosome Integrity and Plasma Membrane Integrity 

A LIVE/DEAD sperm motility test kit (L-7011, Thermo Fisher, Shanghai, China) and fluorescein isothiocyanate–peanut lectin (L-7381, Sigma-Aldrich, Shanghai, China) were used to evaluate the sperm membrane and acrosome integrity. To detect the acrosome integrity, a pure methanol solution was used to fix the sperm samples, followed by 30 min of incubation in the dark with 100 µg/mL fluorescein isothiocyanate–peanut lectin solution and 2.4 mM propidium iodide (PI) solution. To detect the membrane integrity, sperm samples were incubated for a duration of 10 min in the dark with a 100 nM SYBR-14 (Thermo Fisher, Shanghai, China, L7011) working solution and 2.4 mM PI solution. As described by Zhang et al. [35], the stained sperm were observed under a microscope, and images were taken using 400× magnification (ZEISS DM200LED, Oberkochen, Germany) to detect the acrosome integrity and membrane integrity (emitting green fluorescence at 516 nm and red fluorescence at 617 nm). Analyses were carried out in triplicate.

### 2.6. Measurement of Amino Acid Content

According to Schilling et al. [38], accurately weigh 2–5 g of the seminal plasma in a 15 mL centrifuge tube, add 0.01 mol/L of 5 mL of hydrochloric acid, swirl it for 5 min, extract it by ultrasound for 5 min, stand for 2 h away from light, centrifuge at 4000 rpm for 10 min, accurately take 1 mL of supernatant, add 1 mL of 6–8% sulfonyl salicylic acid, swirl for 1 min, stand for 1 h away from light, centrifuge at 15,000 rpm for 15 min, and then take the supernatant and filter it with a 0.22 µm filter membrane. Finally, a high-speed amino acid analyzer (Thermo Fisher, Waltham, MA, USA, 02454, UItimate 3000) was used to detect proline at 440 nm and other amino acids at 570 nm. Analyses were carried out in triplicate.

### 2.7. Measurement of Sperm GSH Content

With the aid of a glutathione assay kit (A061-1, Nanjing Jiancheng Bioengineering Institute, Wuhan, China), the sperm GSH content was measured according to Zhu et al. [39]. Sperm samples were homogenized and centrifugated at 3500 rpm/s, and the supernatant was collected. Thereafter, according to the manufacturer’s instructions, reagents were added to the supernatant and mixed. Following this, a microplate reader was used to take the absorbance (A1) measurement at 532 nm after waiting for 30 s. After 10 min at room temperature, a second absorbance (A2) measurement was carried out. According to the manufacturer’s instructions, reagents were added to the supernatant and mixed prior to incubation for 30 min at 37 °C. The addition of reagents and mixing of the sample were performed again following incubation. Then, the absorbances (A1 and A2) were taken using a microplate reader at 450 nm. Total T-GSH content was obtained by the formula T-GSH − 2 × GSSG. Analyses were carried out in triplicate.

### 2.8. Measurement of Sperm CAT Activities

CAT activity was measured using a catalase assay kit (A007-1-1, Nanjing Jiancheng Bioengineering Institute, Wuhan, China) as previously described by Zhang et al. [35]. Sperm sample homogenate was subjected to centrifugation for 10 min at 3500 rpm/min, and the supernatant was collected. Then, reagents were added to the supernatant and mixed according to the manufacturer’s instructions. After accurately reflecting at 37 °C for 1 min, the absorbance was taken at 405 nm according to the instructions. Analyses were carried out in triplicate.

### 2.9. Measurement of Sperm MDA Content

Sperm MDA content was measured using an MDA assay kit (S0131S, Beyotime Institute of Biotechnology, Shanghai, China) as described by Zhang et al. [40]. Sperm samples were removed from 4 °C and placed on ice, where they were lysed by sonication [41] (20 kHz, 300 W, operating at 50% for 3 min at the rate of 10 s On and 5 s Off) (Thermo Fisher Science, US, FB50220). A pre-prepared reaction buffer reagent was then added to the lysed sample and mixed and boiled for 40 min. Thereafter, the mixture was allowed to cool before centrifugation and the collection of supernatants. A microplate reader (TECAN, Infinite M Nano, Männedorf, Switzerland) was used to take the absorbance readings at 532 nm. Analyses were carried out in triplicate.

### 2.10. Measurement of Testosterone Content

According to the manufacturer’s instruction, an ELISA kit assay [42] (F72025-B, Fankew, Shanghai, China) was used to measure the concentration of testosterone in the ram seminal plasma. An amount of 50 μL of each sample with 100 μL of testosterone-labeled horseradish peroxidase (HRP), the control, and the calibrator were loaded in duplicate in a microtiter plate coated with an anti-testosterone-specific antibody. Then, the microtiter plate containing the samples was incubated at room temperature. The microtiter plate wells were then washed three times at the completion of the incubation period (60 min). Following the addition of 100 μL of chromogenic substrate (TMB) to each well, the plates were incubated at room temperature away from a light source. After a 30 min incubation, 100 μL of 0.2 M HCl solution was added to each well, and absorbance readings at 450 nm were taken using a microtiter plate reader. Analyses were carried out in triplicate.

### 2.11. Measurement of FSH Content

The FSH level in THE serum was measured by a FSH assay kit (Mlbio, Shanghai, China, ml061713) [43]. Briefly, a specific anti-sheep FSH antibody was pre-coated on a high-affinity microplate. The standards and samples were then added to the wells and incubated. During the incubation, the samples’ FSH were bound to the solid-phase antibody. After washing to remove unbound substances, a biotinylated detection antibody was added. Thereafter, it was washed to remove the unbound biotinylated antibody, and then the streptavidin was conjugated to horseradish peroxidase (Streptavidin-HRP). After that, a TMB substrate was added and measured. The intensity of the color observed determined the concentration of FSH in the sample. Thereafter, a stop solution was added to terminate the reaction. Then, absorbance readings were taken at 450 nm with a reference wavelength of 570–630 nm using a microplate reader. Analyses were carried out in triplicate.

### 2.12. Measurement of LH Content

The LH level in the serum was measured by an LH assay kit (Mlbio, China, ml061715) [44]. The microplate was pre-coated with an anti-sheep LH antibody. During the experiment, ram LH in the samples or standards bound to the coated antibody. Subsequently, a biotinylated anti-sheep LH antibody and streptavidin conjugated to horseradish peroxidase (HRP) were added. The biotinylated antibody bound to the captured sheep LH, and streptavidin specifically bound to the biotin, forming an immune complex. Unbound components were washed away. A TMB substrate which turns blue under the catalytic action of HRP and yellow in the presence of a stop solution was added. The optical density (OD) was taken at 450 nm, with the concentration of LH being directly proportional to the OD450 value. The LH concentration in the samples was calculated by plotting a standard curve. Analyses were carried out in triplicate.

### 2.13. Seminal Plasma Metabolite Separation

According to a previous study [45], the seminal plasma samples collected were cooled on ice before vortexing for 30 s for proper mixing. Then, ice-cold methanol was added to seminal plasma in a 3 to 1 ratio by volume. Following that, the sample was vortexed for 3 min and centrifuged (4 °C) for 10 min at 12,000× *g*. Thereafter, the supernatant collected was re-centrifuged for 5 min at the same setting. Following that, the supernatants were filtered using a 0.22 µm membrane, and then the filtrates were placed in injection bottles. Samples collected were stored in −80 °C before LC-MS/MS analysis. In addition, the pooled Quality Control (QC) samples were simultaneously prepared by mixing 10 μL of each exacted mixture.

### 2.14. UHPLC-MS/MS

According to Keguang Han et al. [46], a UPLC-ESI-Q-Orbitrap-MS system (UHPLC, Shimadzu Nexera X2 LC-30AD, Shimadzu, Kyoto, Japan) coupled with Q-Exactive Plus (Thermo Fisher, San Jose, CA, USA) was used for the metabolomic profiling analysis.

An ACQUITY UPLC^®^ HSS T3 column (2.1 × 100 mm^2^, 1.8 μm) (Waters, Milford, MA, USA) was used to analyze samples for liquid chromatography (LC) separation. Constituents of the mobile phase included A: 0.1% FA in water and B: 100% acetonitrile (ACN), while the flow rate was set at 0.3 mL/min. The gradient was at 0% buffer B for 2 min and was linearly increased to 48% in 4 min, then up to 100% in 4 min and maintained for 2 min, and then decreased to 0% buffer B in 0.1 min, with a 3 min re-equilibration period employed.

The electrospray ionization (ESI) with both negative and positive was utilized for the MS data acquisition separately. The set ESI source conditions were as follows: spray voltages: 3.8 kv (positive) and 3.2 kv (negative); capillary temperature: 320 °C; sheath gas (nitrogen) flow: 30 arb (arbitrary units); aux gas flow: 5 arb; probe heater temp.: 350 °C; S-Lens RF level: 50. To achieve full MS, the instrument was set to acquire over the m/z range 70–1050 Da. Full MS scans were obtained at resolutions of 70,000 at *m*/*z* 200 and 17,500 at *m*/*z* 200 for the MS/MS scan. For the MS and MS/MS, the maximum injection times were set to 100 ms and 50 ms, respectively. More so, while the normalized collision energy (stepped) was set at 20, 30, and 40 for fragmentation, the isolation window for MS2 was set to 2 *m*/*z*.

### 2.15. Data Preprocessing and Filtering

MS-DIAL was used to process raw MS data for retention time correction, peak alignment, and peak area extraction. The accuracy mass (mass tolerance < 10 ppm) and MS/MS data (mass tolerance < 0.02 Da), which were matched with HMDB, mass bank, our self-built metabolite standard library, and other public databases, were used in identifying metabolites. More so, only variables with over 50% of nonzero measurement values in at least one group were kept in the extracted-ion features.

### 2.16. Multivariate Statistical Analysis

All multivariate data analyses and modeling were performed using R software (version 4.0.3) and R packages. Data were mean-centered by Pareto scaling. Models were built based on partial least-square discriminant analysis (PLS-DA), principal component analysis (PCA), and orthogonal partial least-square discriminant analysis (OPLS-DA). All the models evaluated were tested for overfitting with the methods of permutation tests. R2X (cumulative) (perfect model: R2X (cum) = 1) and R2Y (cumulative) (perfect model: R2Y (cum) = 1) values were used to determine the descriptive performances of the models. Likewise, their prediction performances were measured by Q2 (cumulative) (perfect model: Q2 (cum) = 1) and a permutation test (*n* = 200). The permuted model should not be able to predict classes: the R2 and Q2 values at the *Y*-axis intercept must be lower than those of the Q2 and R2 of the non-permuted model. OPLS-DA is used for determining discriminating metabolites utilizing the variable importance on projection (VIP). The VIP score value indicates a variable’s contribution to the discrimination between all sample classes. These aforementioned scores are obtained for each variable as a weighted sum of squares of the PLS weights. The mean VIP value is 1, and, usually, VIP values that are more than 1 are said to be significant. A high score is associated with a strong discriminatory ability and thus constitutes a criterion for the selection of biomarkers.

The discriminating metabolites were calculated using a statistically significant threshold of the variable influence on projection (VIP) values. These values were obtained from the OPLS-DA model and two-tailed Student’s *t*-test (*p*-value) on the normalized raw data at the univariate analysis level. In addition, one-way analysis of variance (ANOVA) for multiple-group analysis was used to calculate the *p*-value. Metabolites are deemed to be statistically significant when their *p* < 0.05 and VIP > 1.0. The logarithm of the average mass response (area) ratio between two arbitrary classes is the fold change. In addition, the identified differential metabolites were utilized in carrying out cluster analyses with the R package.

### 2.17. KEGG Enrichment Analysis

The differential metabolite data were subjected to KEGG pathway analysis using the KEGG database (http://www.kegg.jp, accessed on 24 March 2023) to identify the perturbed biological pathways. KEGG enrichment analyses were performed using Fisher’s exact test, and FDR correction for multiple tests was also conducted. At a *p*-value less than 0.05, enriched KEGG pathways were deemed to be nominally statistically significant.

### 2.18. Statistical Analysis

All data were analyzed by one-way ANOVA, and Tukey’s multiple-comparison test was performed using SPSS version 26.0 for Windows (SPSS Inc, Chicago, IL, USA). All values are presented as mean ± standard error of the mean (SEM). Differences with values of *p* < 0.05 were statistically significant.

## 3. Results

### 3.1. Extended Light Duration Improved Sperm Motility Parameters

As shown in Table 1, CASA analysis shows that the sperm total motility, progressive motility, as well as the VCL, VSL, VAP, STR, WOB, and LIN, were increased (*p* < 0.05) by the artificial extension of the light duration (14 h, 16 h, 18 h) when compared to those in the control. Specifically, the 16 h light treatment had the highest value for the above-mentioned parameters (*p* < 0.05) among all the treatments. Regarding the ALH and BCF, there were no differences (*p* > 0.05) among the treatments after extended light duration processing. Moreover, there was no significant difference in the sperm motility parameters between the 20 h light treatment group and the control group.

### 3.2. Extended Light Duration Improved the Sperm Abnormality, Concentration, Semen Volume, and Total Sperm Number

**Figure 1:**
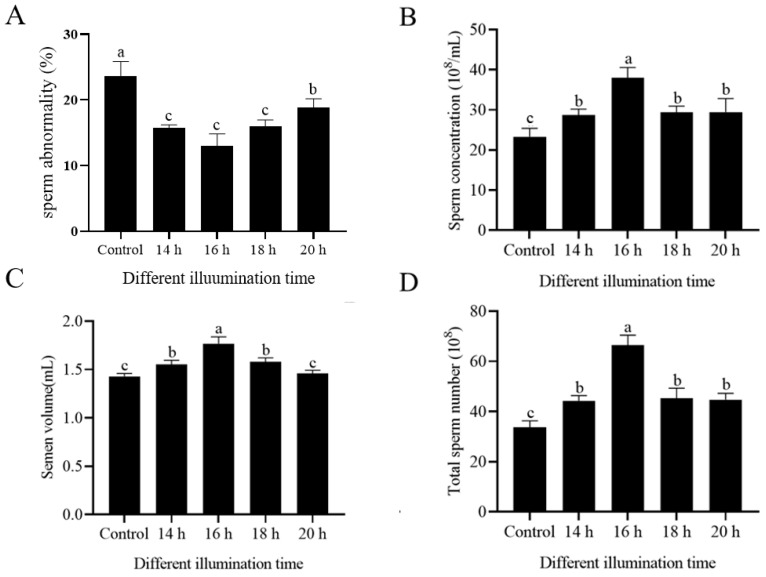
Effects of extended light duration on sperm abnormality (**A**), concentration (**B**), semen volume (**C**), and total sperm number (**D**) of sheep sperm. Values are presented as mean ± standard error of the mean (SEM). Columns with different lowercase letters were significantly different (*p* < 0.05), n = 3.

As shown in Figure 1A, the extended light duration reduced the rate of sperm abnormality compared with the control group (*p* < 0.05). The reduction in sperm abnormality was particularly enhanced in the 16 h light group (*p* < 0.05). Moreover, it can be seen from Figure 1B–D that compared with the control group, the extended light duration improved the sperm ejaculation volume, sperm concentration, and total sperm number. Similarly, the effect of the 16 h light group is obvious (*p* < 0.05). As shown in Figure 1C, the sperm volume was significantly increased after the 14–18 h light duration, and there was no statistical significance between the 20 h light group and the control group (*p* > 0.05).

### 3.3. Extended Light Duration Improved Sperm Acrosome Integrity and Plasma Membrane Integrity

**Figure 2:**
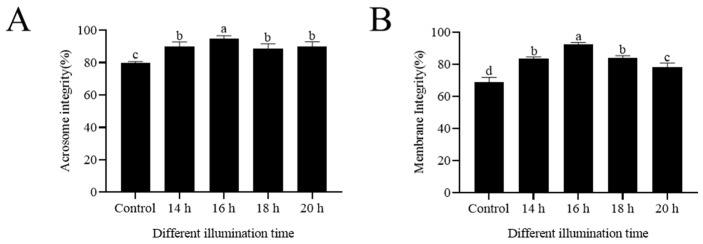
Effects of extended light duration on acrosome integrity (**A**) and membrane integrity (**B**) of sheep sperm. Values are presented as mean ± standard error of the mean (SEM). Columns with different lowercase letters were significantly different (*p* < 0.05), n = 3.

The artificial extension of the light duration (14 h, 16 h, 18 h, 20 h) greatly promoted the acrosomal integrity of the sperm (*p* < 0.05) (Figure 2A). The 16 h light group showed more intact acrosomes than the control group (*p* < 0.05) (Figure 2A). In addition, the artificial extension of the light duration improved the sperm plasma membrane integrity (*p* < 0.05), and the highest values in this parameter were observed in the 16 h light group (Figure 2B).

### 3.4. Extended Light Duration Enhanced the Concentration of Free Amino Acids

Compared with the control group, the total amino acid contents of the seminal plasma in the 16 h light group were increased (*p* < 0.05) (Table 2). Among them, the glutamic acid content was the most abundant, and tyrosine was the least. Interestingly, however, the contents of methionine (Met) and tyrosine (Tyr) in the seminal plasma was not different between the 16 h light group and the control group (*p* > 0.05) (Table 2).

### 3.5. Extended Light Duration Enhanced the Concentrations of Testosterone, FSH, and LH

**Figure 3:**
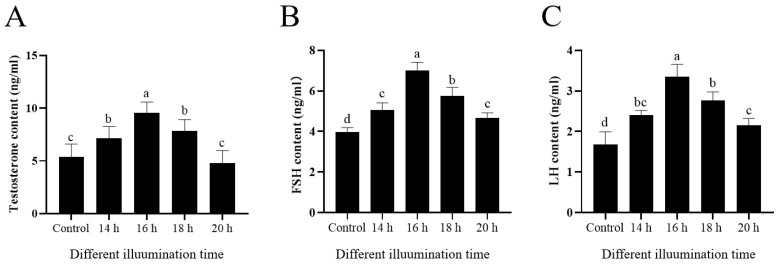
Effects of different light durations on testosterone content (**A**), serum FSH content (**B**), and serum LH content (**C**). Values are presented as mean ± standard error of the mean (SEM). Columns with different lowercase letters were significantly different (*p* < 0.05), n = 3.

As shown in Figure 3A, compared with the control group, the testosterone content in the seminal plasma of the 16 h light group was significantly increased (*p* < 0.05), while the 20 h light treatment decreased it.

As shown in Figure 3B, it was observed that the extension of the light duration (14 h, 16 h, 18 h, 20 h) increased the ram serum FSH levels (*p* < 0.05) when compared to the control. And the 16 h light group showed the highest value among all the treatments (Figure 3B). Interestingly, the LH levels in the groups with 14 h,16 h, 18 h or 20 h of light exposure were higher than those in the control, and the LH level in the 16 h of light exposure treatment showed the highest value among the treatments (Figure 3C).

### 3.6. Extended Light Duration Improved Sperm Antioxidative Stress

**Figure 4:**
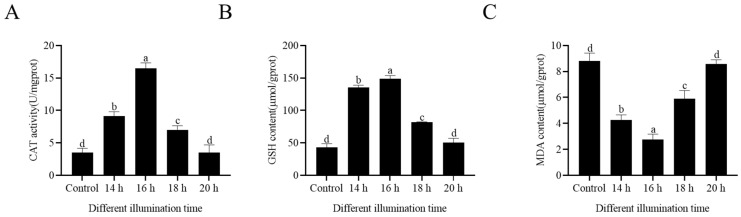
Effects of different light durations on sperm CAT activity (**A**), GSH content (**B**), and MDA content (**C**). Values are presented as mean ± standard error of the mean (SEM). Columns with different lowercase letters were significantly different (*p* < 0.05), n = 3.

The CAT activity, GSH content, and MDA content of the sheep sperm were assessed to investigate the impact of the extended light duration on the sperm’s antioxidant capacity. As shown in Figure 4A, the CAT activity was the highest in the group exposed to 16 h of light. The CAT activity decreased at 18 h of light exposure, and there was no statistically significant difference in the CAT activity between the 20 h light exposure group and the control group (*p* < 0.05). Additionally, within the 14 to 18 h light exposure range, increasing the light exposure time led to an increase in the GSH content (*p* < 0.05), while the 20 h of light exposure resulted in a decrease in the GSH content (Figure 4B). Compared to the control group, the MDA content decreased with prolonged light exposure, with the lowest MDA content observed at 16 h of light exposure (*p* < 0.05). Interestingly, the MDA content increased at 20 h of light exposure, but with no statistical significance compared with the control group (Figure 4C).

### 3.7. Identification of Metabolomic Data

The non-targeted metabolomic strategy, including the positive and negative modes, was employed in the analysis of the ram seminal plasma. The results showed that 1568 metabolites, which included organic acids, amino acids, nucleic acids, peptides, sugars, fatty acids, lipids, and other metabolites, were identified in the seminal plasma of the control group and the 16 h light group. Comprehensive information on these identified metabolites, as well as their retention times (RTs), compounds, indexes, masses, formulae, and other information, can be found in Supplementary Table S1.

### 3.8. Determination of Differential Metabolites

**Figure 5:**
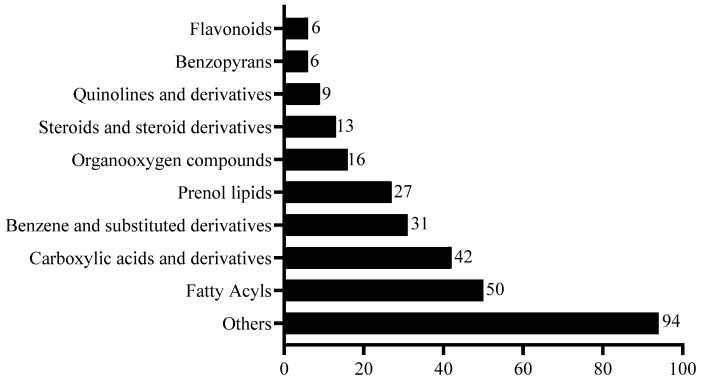
The classification of differential metabolites presented in ram seminal plasma between the 16 h light group and the control group. The number on each column represents the number of that kind of metabolite.

The differential metabolites between the control and 16 h light group were identified on the basis of the following: the variable importance in projection (VIP), fold change (FC), and *p*-value. Comprehensive information on these identified metabolites can be found in Supplementary Table S1. In total, 345 differential metabolites were identified between the 16 h light group and the control group. Among these metabolites, 273 metabolites were upregulated in the 16 h light group. However, 72 metabolites were significantly enriched in the control group. The numbers of the various constituent compounds in the ram seminal plasma are shown in Figure 5.

### 3.9. Bioinformatics Analysis of Differential Metabolites

**Figure 6:**
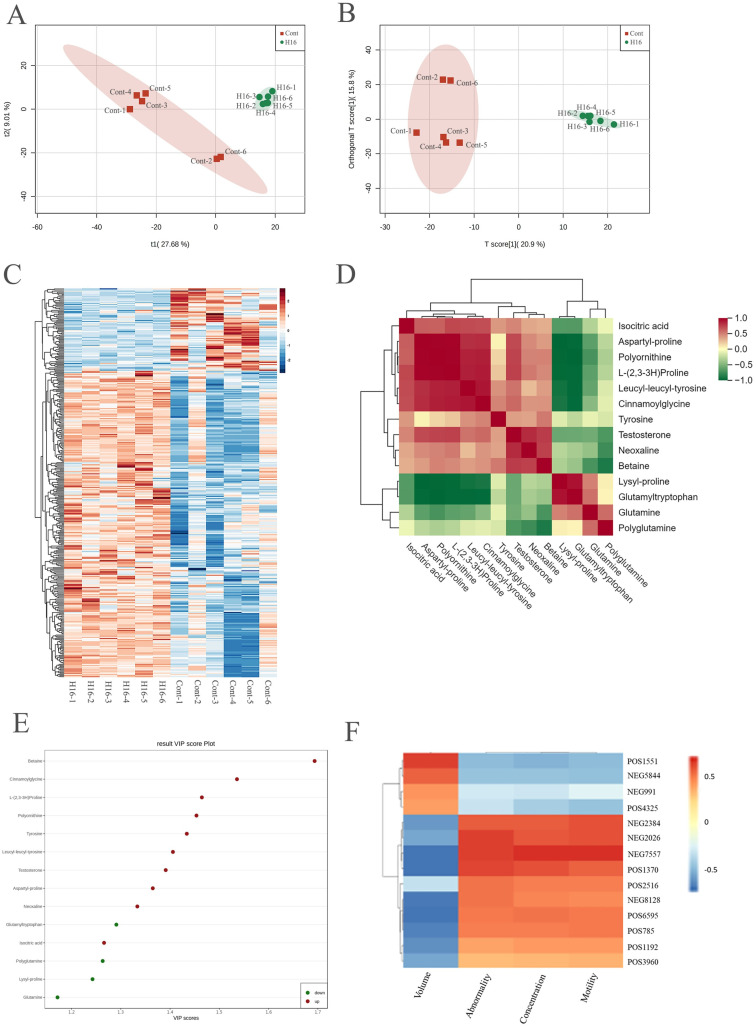
The bioinformatics analysis of differential metabolites between the 16 h light group and the control group. (**A**,**B**) The separation degree between the 16 h light and control groups. (**C**) The clusters heatmap of different metabolites. The metabolites with significant differences were normalized and clustered in this map. The *X*-axis represents the samples, and the *Y*-axis represents the differential metabolites. Red represents the highly expressed metabolites, and green represents the lowly expressed metabolites. (**D**) The correlation results of differential metabolites were analyzed by the Pearson correlation analysis method. The red color indicates a strong positive correlation, and the green color indicates a strong negative correlation. (**E**) The differential metabolites with the VIP values. The abscissa represents the VIP value, and the ordinate represents the differential metabolite. Red represents upregulated metabolites, and green represents downregulated metabolites. (**F**) The correlation between sperm metabolites and sperm concentration, volume, abnormality, and motility.

PLS-DA and OPLS-DA analyses showed that the 16 h light group and control group were well separated (Figure 6A,B). As shown in Figure 6C, the cluster patterns between the 16 h light group and the control group were the opposite. The metabolites enriched in the 16 h light group were less abundant in the control group. Figure 6D shows the correlation analysis results for the differential metabolites obtained using Pearson correlation analysis. Fourteen metabolites associated with semen quality were shown after the analysis. The significant correlations existing among these metabolites were assessed. Aspartyl-proline (POS3960) was negatively correlated with lysyl-proline (POS4325) (correlation coefficient: −0.93) and positively correlated with polyornithine (POS1192) (correlation coefficient: 0.98).

The 14 differential metabolites with their VIP values are shown in Figure 6E. These metabolites are as follows: glutamyltryptophan (NEG 5844), polyglutamine (POS1551), lysyl-proline (POS4325), and glutamine (NEG991), which were enriched in the low-motility group, and leucyl-leucyl-tyrosine (NEG7557), polyornithine (POS1192), testosterone (POS6595), cinnamoylglycine (NEG2384), neoxaline (NEG8128), aspartyl-proline (POS3960), l-(2,3-3H) proline (POS785), betaine (POS1370), isocitric acid (NEG2026), and tyrosine (POS2516), which were more abundant in the high-motility group. These 14 metabolites were well correlated with the sperm motility, sperm concentration, sperm volume, and sperm abnormality (Figure 6F), with the increased metabolites in the 16 h light group being positively correlated with these sperm parameters, while the decreased metabolites in the 16 h light group were negatively correlated.

### 3.10. Functional Annotation of Differential Metabolites

**Figure 7:**
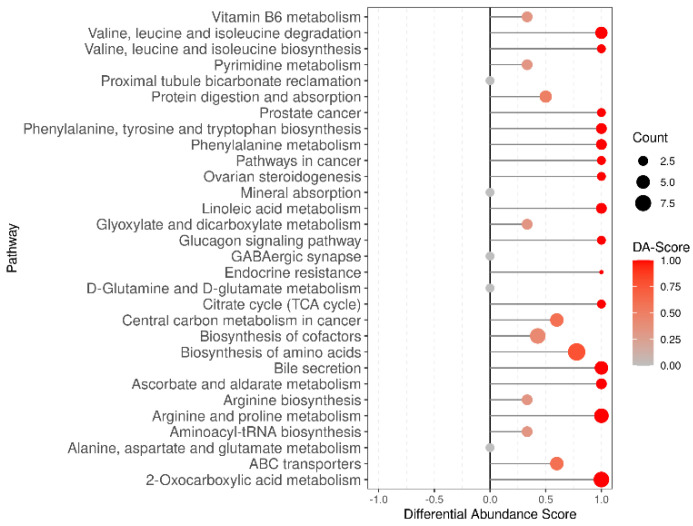
The KEGG enrichment map of differential metabolites. Abscissa represents the Rich factor, and the ordinate represents the pathway name. The color of the point represents the *p*-value. Red indicates that the enrichment is more significant. The sizes of the points represent the numbers of metabolites involved in this pathway.

All the potential pathways that the acquired differential metabolites may be associated with were obtained with the aid of KEGG annotation. As shown in Figure 7, the metabolites identified were involved in metabolic and synthetic activities. These metabolic activities included valine, leucine, and isoleucine degradation; valine, leucine, and isoleucine biosynthesis; protein digestion and absorption; phenylalanine, tyrosine, and tryptophan biosynthesis; phenylalanine metabolism; the citrate cycle (TCA cycle); the biosynthesis of amino acids; aminoacyl−tRNA biosynthesis; aspartate and glutamate metabolism. Comprehensive information on this can be found in Supplementary Table S2.

## 4. Discussion

Seasonal variations regulate the sperm quality parameters, including the sperm motility, ejaculation volume, and sperm density in sheep as a seasonal breeding animal [47]. The underlying mechanism of this phenomenon involves a complex interplay between endogenous periodic light rhythms and synchronization [44]. The manipulation of artificial photoperiods in sheep, which involves natural light and extended daylight supplemented with additional illumination, is widely employed to synchronize the breeding season according to the requirements of animal producers or to effectively mitigate seasonal variations in sperm production at artificial insemination centers [48]. The implementation of melatonin-free pure light treatment, particularly in open barns, can be regarded as a non-invasive approach that fully upholds animal welfare principles [48].

Sperm motility parameters such as the total and progressive motility and plasma membrane and acrosomal integrities are essential to the sperm penetration of the zona pellucida and the completion of conception after artificial insemination [49]. In this study, the total motility, progressive motility, VCL, VSL, VAP, LIN, WOB, plasma membrane integrity, and acrosome integrity in the 16 h light group were significantly higher than those in the control group. In addition, the ejaculation volume and sperm density increased significantly in the 16 h light group compared with the control group. It has been reported that after the extension of the winter light duration, there was a significant improvement in the sperm quality [50]. This aligns with the results in this study showing that prolonging the photoperiod improved the sperm quality. Interestingly, however, El-Alamy et al. [48] reported that the ejaculation volume was not affected in Finnish rams after artificially setting the photocycle but was affected in Dorset rams. From this, the effect of the photoperiod on the ejaculation volume may be influenced by both high- and low-sex drive sheep breeds.

Interestingly, Yasuo et al. [51] reported that compared to the control, long-day stimulus decreased the testicular goat weight by 70%. This is slightly different from our experimental conclusion. In this study, increasing the light duration to 16 h significantly increased the testosterone levels, beneficial seminal plasma components, and oxidative stress-related enzyme (CAT, GSH, and MDA) activities compared to the control group. All these factors are associated with the sperm quality in different aspects. Moreover, steroids and their derivative steroid hormones are essential for sexual organ development, spermatogenesis, and sperm quality. In this study, 13 steroid hormones, including testosterone, estradiol, progesterone, cortisol, corticosterone, dehydroepiandrosterone, androstenedione, 17α-hydroxyprogesterone, 11-deoxycorticosterone, aldosterone, dihydrotestosterone, estrone, and pregnenolone, were identified. Among them, testosterone was significantly increased in the 16 h light group. Testosterone plays a series of important roles in male physiology, promoting sperm maturation in the epididymis by stimulating androgens on the supporting cells in the spermatogenic tubules and maintaining spermatogenesis. Interestingly, testosterone is essentially the final product of the hypothalamic–pituitary–gonadal axis in male mammals. Following an increase in testosterone secreted by Leydig cells, there is a negative-feedback inhibition on the hypothalamus to reduce the secretion of GnRH, which, in turn, suppresses the secretion of FSH and LH from the anterior pituitary. LH acts on Leydig cells, promoting the secretion of testosterone [52], while FSH acts on Sertoli cells, triggering a series of physiological effects. The most crucial effect of FSH is inducing Sertoli cells to secrete androgen-binding protein (ABP), which binds to testosterone produced by Leydig cells and transports it to the vicinity of spermatogenic cells in the seminiferous tubules, thereby initiating spermatogenesis [53,54]. In fact, in this experiment, both the FSH and LH levels significantly increased under 16 h of light exposure, further confirming that, in male mammals, spermatogenesis and steroidogenesis are largely dependent on the gonadotropin levels. The reason for the slight difference between the two studies may be that the sheep in this study were not typical short-day breeders. Moreover, in this study, the sheep were housed under LED-light exposure, which is different than the natural light used in the report of Yasuo et al. [45], which might be one of the reasons for the different results between our study and that of Yasuo et al. [45]. So, to be consistent with this, we need to further investigate the effects of different light types on sperm, the breed-specific responses to varying light durations, and the role of specific metabolites in sperm protection under different photoperiods in the future. Moreover, in this study, the change in the hormone levels could have been induced by the varying light durations, suggesting that artificial photoperiods might be beneficial for animal welfare, as the stress or behavioral changes resulted from melatonin suppression.

Many components of seminal plasma play an important role in male fertility and sperm function. By non-targeted metabolomic assessment, 42 carboxylic acids and their derivatives were identified in the seminal plasma with significant differences between the control and 16 h light groups. Among them, amino acids in carboxylic acid metabolism and their derivatives are widely involved in the protection and regulation of metabolic activities and sperm biology [55]. Amino acids also protect sperm by reducing sperm damage caused by lipid peroxidation and free radicals. The present study revealed that the CAT activity and GSH content were significantly increased, and the MDA content of the ram sperm subjected to 16 h of light exposure was significantly lowered, compared to those in the control. Changes in amino acids, antioxidants, and tricarboxylic acid (TCA)-cycle intermediates play critical roles in sperm function by influencing energy production, oxidative stress management, and cellular signaling [56]. Amino acids like glutamine and alanine serve as energy substrates, fueling the ATP production necessary for sperm motility [56]. Catalase [57], superoxide dismutase [58], and glutathione peroxidase [59] in seminal plasma protect sperm membranes from peroxidation. The TCA cycle is central to ATP generation in sperm mitochondria, which power flagellar movement and thus motility [60]. Alterations in TCA intermediates can affect sperm functionality, such as citrate and malate, because they can enhance ATP synthesis to directly affect motility and endurance [61]. Isocitric acid, a key intermediate in the TCA cycle, was also significantly increased in the 16 h light group in the present study. A previous study reported that isocitric acid can activate isocitrate dehydrogenase 3b (IDH3), and the inactivation of IDH3 could lead to the insufficient energy of the acrosomes and flagella in sperm [62]. In this study, the metabolome database of ovine seminal plasma was established for different light durations, and the metabolites that may be involved in the regulation of sperm motility under the influence of light were identified. A total of 346 different metabolites were found between the natural light and 16 h light groups, and 273 increased metabolites were found in the 16 h light group. In addition, the KEGG analysis demonstrated that the differential metabolites were associated with amino acid metabolism and the tricarboxylic acid cycle, indicating that the 16 h light treatment improved the ram sperm motility by the increase in seminal plasma metabolites which enter the TCA for ATP generation. Therefore, the metabolites obtained in this study may help to further understand the mechanism of the effect of light on sperm motility.

## 5. Conclusions

In this study, the artificial extension of the light duration, especially for 16 h, with the type of light source (LED; warm white light; wavelength: 2800–3200 nm; brightness: 400–600 mcd) improved the HongDe ram line sperm characteristics, testosterone levels, antioxidant enzyme activities, and seminal plasma metabolomics in the rams. Thus, photoperiod manipulation is a beneficial tool for developing sheep artificial insemination.

## Figures and Tables

**Table 1:** The sperm parameters of the control group and different light groups were analyzed by CASA.

Sperm Parameters	Control	14 h	16 h	18 h	20 h
Total motility (%)	76.5 ± 2.36 ^c^	89.27 ± 0.84 ^b^	95.2 ± 2.17 ^a^	92.07 ± 1.63 ^ab^	85.23 ± 3.80 ^bc^
Progressive motility (%)	49.03 ± 5.35 ^c^	76.72 ± 4.36 ^ab^	83.50 ± 3.10 ^a^	72.77 ± 6.15 ^b^	56.97 ± 1.59 ^c^
VCL (μm/s)	63.25 ± 4.96 ^c^	85.71 ± 9.52 ^b^	108.75 ± 5.33 ^a^	90.99± 4.96 ^b^	66.86 ± 1.61 ^c^
VSL (μm/s)	30.72 ± 2.13 ^c^	57.38 ± 10.32 ^ab^	67.89 ± 8.07 ^a^	50.40 ± 2.16 ^b^	31.49 ± 4.16 ^c^
VAP (μm/s)	39.65 ± 3.40 ^c^	66.30 ± 10.58 ^b^	81.38 ± 8.37 ^a^	63.43 ± 2.44 ^b^	40.44 ± 2.92 ^c^
BCF (Hz)	6.59 ± 0.61	7.94 ± 2.30	7.12 ± 0.44	6.33 ± 0.68	7.18 ± 1.27
ALH (μm)	3.57 ± 0.06	3.44 ± 0.13	4.49 ± 0.30	4.47 ± 0.06	3.63 ± 0.41
STR (%)	77.56 ± 1.24 ^c^	86.37 ± 3.36 ^a^	83.32 ± 1.54 ^a^	79.46 ± 0.35 ^bc^	77.64 ± 4.56 ^c^
LIN (%)	48.60 ± 1.52 ^c^	66.61 ± 4.67 ^a^	62.31 ± 5.11 ^ab^	55.43 ± 1.19 ^bc^	47.18 ± 7.18 ^c^
WOB (%)	62.68 ± 2.14 ^c^	77.11 ± 4.33 ^ab^	74.73 ± 4.78 ^a^	69.76 ± 1.56 ^bc^	60.56 ± 5.58 ^c^

Values are expressed as mean ± standard error. Different letters within the same row indicate significant differences (*p* < 0.05), n = 3. VCL, curvilinear velocity; VSL, straight-line velocity; VAP, average path velocity; BCF, beat-cross frequency; ALH, lateral head; STR, straightness (VSL/VAP); LIN, linearity (VSL/VCL); WOB, wobble (VAP/VCL).

**Table 2:** Amino acid contents in seminal plasma of the control group and 16 h light group.

Amino Acid (μmol/L)	Control	16 h
Aspartic Acid (Asp)	239.05 ± 1.41 ^b^	402.37 ± 1.98 ^a^
Threonine (Thr)	138.84 ± 1.82 ^b^	273.54 ±1.53 ^a^
Serine (Ser)	145.88 ±1.03 ^b^	311.38 ±4.10 ^a^
Glutamate (Glu)	2458.68 ± 15.94 ^b^	4924.65 ±17.52 ^a^
Glycine (Gly)	463.14 ±5.08 ^b^	1471.27 ±8.77 ^a^
Alanine (Ala)	185.12 ±2.12 ^b^	468.96 ±3.29 ^a^
Cysteine (Cys)	41.22 ±6.92 ^b^	58.00 ±5.11 ^a^
Valine (Val)	104.26 ±5.96 ^b^	295.32 ±5.74 ^a^
Methionine (Met)	20.73 ±2.50	13.09 ±1.64
Isoleucine (Ile)	45.19 ±0.33 ^b^	113.85± 0.25 ^a^
Leucine (Leu)	69.84 ±0.63 ^b^	180.44 ±1.38 ^a^
Tyrosine (Tyr)	5.12 ±0.17	6.07 ±0.75
Phenylalanine (Phe)	51.68 ±9.46 ^b^	104.45 ±7.67 ^a^
Lysine (Lys)	79.80 ±1.72 ^b^	174.89 ±3.0 ^a^
Histidine (His)	35.45 ±14.24 ^b^	50.39 ±14.40 ^a^
Arginine (Arg)	34.65 ±0.26 ^b^	85.11 ±0.85 ^a^
Proline (Pro)	176.64 ± 16.19 ^b^	346.46 ± 22.54 ^a^

Values are expressed as mean ± standard error. Different letters within the same row indicate significant differences (*p* < 0.05), n = 3.

## Data Availability

The data presented in this study are available in the article.

## Supplementary Materials

The following supporting information can be downloaded at https://www.mdpi.com/article/10.3390/ani14243592/s1, Table S1: comprehensive information on the identified metabolites. Table S2: comprehensive information on functional annotation of differential metabolites.
